# Increased high molecular weight adiponectin and lean mass during tocilizumab treatment in patients with rheumatoid arthritis: a 12-month multicentre study

**DOI:** 10.1186/s13075-020-02297-7

**Published:** 2020-09-29

**Authors:** Eric Toussirot, Hubert Marotte, Denis Mulleman, Grégoire Cormier, Fabienne Coury, Philippe Gaudin, Emmanuelle Dernis, Christine Bonnet, Richard Damade, Jean-Luc Grauer, Tassadit Ait Abdesselam, Caroline Guillibert-Karras, Frédéric Lioté, Pascal Hilliquin, Antoinette Sacchi, Daniel Wendling, Benoît Le Goff, Marc Puyraveau, Gilles Dumoulin

**Affiliations:** 1 INSERM CIC-1431, CHU de Besançon, Centre d’Investigation Clinique Biothérapie, Pôle Recherche, 25000 Besançon, France; 2 Fédération Hospitalo-Universitaire INCREASE, CHU de Besançon, 25000 Besançon, France; 3 CHU de Besançon, Rhumatologie, Pôle PACTE (Pathologies Aiguës Chroniques Transplantation Éducation), 25000 Besançon, France; 4 Université de Bourgogne Franche-Comté, Département Universitaire de Thérapeutique, Besançon, France; 5 INSERM UMR1098 « Relations Hôte Greffon Tumeurs, ingénierie cellulaire et génique », Université de Bourgogne Franche-Comté, 25000 Besançon, France; 6 INSERM 1059, Université de Lyon, Saint-Etienne; Rhumatologie CHU de Saint-Etienne; CIC-1408, CHU de Saint-Etienne, Saint-Etienne, France; 7 Rhumatologie CHU de Tours, Tours, France; 8 Rhumatologie Centre Hospitalier Départemental Vendée, La Roche sur Yon, France; 9 Rhumatologie Hospices Civils de Lyon, INSERM UMR1033, Université Lyon 1, Lyon, France; 10 Rhumatologie CHU de Grenoble, Grenoble, France; 11 Rhumatologie Centre Hospitalier, Le Mans, France; 12 Rhumatologie CHU de Limoges, Limoges, France; 13 Rhumatologie Centre Hospitalier, Chartres, France; 14 Rhumatologie Centre Hospitalier, Montélimar, France; 15 Rhumatologie Centre Hospitalier, Meaux, France; 16 Rhumatologie Hôpital St Joseph, Marseille, France; 17 Rhumatologie Hôpital Lariboisière AP-HP Paris, Université de Paris, Paris, France; 18 Rhumatologie Centre Hospitalier Sud Francilien, Corbeil-Essonnes, France; 19 Rhumatologie Centre hospitalier Mantes la Jolie, Mantes-la-Jolie, France; 20 Rhumatologie CHU de Nantes, Nantes, France; 21 Unité de méthodologie uMETh, INSERM CIC-1431, Centre d’Investigation Clinique, CHU de Besançon, Besançon, France; 22 Laboratoire de Biochimie Médicale, UF de Biochimie Endocrinienne et Métabolique, CHU de Besançon; EA 3920 Marqueurs pronostiques et facteurs de régulation des pathologies cardiaques et vasculaires, Université de Bourgogne Franche Comté, Besançon, France

**Keywords:** Adiponectin, Cardiovascular risk, Rheumatoid arthritis, Tocilizumab, Body composition

## Abstract

**Background:**

Patients with rheumatoid arthritis (RA) have an increased risk of cardiovascular (CV) disease. Adiponectin is involved in the metabolism of glucose and lipids with favourable effects on CV disease, especially its high molecular weight (HMW) isoform. Body composition changes are described in RA with various phenotypes including obesity. The effects of tocilizumab on serum adiponectin and body composition, especially fat mass, in patients with RA are not well determined.

**Methods:**

Patients with active RA despite previous csDMARDs and/or bDMARDs and who were tocilizumab naïve were enrolled in a multicentre open-label study. They were evaluated at baseline, 1, 3, 6 and 12 months. Clinical assessment included body mass index (BMI) and anthropometric measurements. Lipid and metabolic parameters, serum adiponectin (total and HMW), leptin, resistin and ghrelin were measured at each time point. Body composition (lean mass, fat mass, % fat, fat in the android and gynoid regions) was evaluated at baseline, 6 and 12 months.

**Results:**

One hundred seven patients were included. Both total and HMW adiponectin significantly increased from baseline to month 3, peaking respectively at month 3 (*p* = 0.0105) and month 1 (*p* < 0.0001), then declining progressively until month 6 to 12 and returning to baseline values. Significant elevation in HMW adiponectin persisted at month 6 (*p* = 0.001). BMI and waist circumference significantly increased at month 6 and 12, as well as lean mass at month 6 (*p* = 0.0097). Fat mass, percentage fat and android fat did not change over the study period. Lipid parameters (total cholesterol and LDL cholesterol) increased while glycaemia, insulin and HOMA-IR remained stable. Serum leptin, resistin and ghrelin did not change during follow-up.

**Conclusions:**

Tocilizumab treatment in RA patients was associated with a significant increase in total and HMW adiponectin, especially at the onset of the treatment. Tocilizumab also induced a significant gain in lean mass, while fat mass did not change. These variations in adiponectin levels during tocilizumab treatment could have positive effects on the CV risk of RA patients. In addition, tocilizumab may have an anabolic impact on lean mass/skeletal muscle.

**Trial registration:**

The ADIPRAT study was a phase IV open-label multicentre study retrospectively registered on ClinicalTrials.gov under the number NCT02843789 (date of registration: July 26, 2016).

## Introduction

Rheumatoid arthritis (RA) is a chronic inflammatory joint disease, leading to progressive clinical deformation, erosive radiographic changes and disability. The mechanisms associated with this chronic inflammation involve different cellular subsets, but also well-identified inflammatory mediators including chemokines and cytokines. Pro-inflammatory cytokines such as IL-1β, TNFα and IL-6, but also IL-17A play a major role in joint inflammation in RA [1]. It is well known that patients with RA have an increased risk of mortality and morbidity from cardiovascular (CV) disease [2, 3]. Indeed, RA is considered to be a risk factor for CV disease, equal to that of type 2 diabetes mellitus [4]. It is considered that the combination of traditional CV risk factors such as smoking, high blood pressure, diabetes and dyslipidaemia, together with systemic inflammation, may explain the increased CV morbidity in RA [2, 3]. Systemic inflammation is a key determinant of accelerated atherosclerosis, while C-reactive protein (CRP), an acute-phase reactant commonly measured in practice, is associated with atherosclerosis, CV risk and mortality [5, 6]. The relationships between lipids and inflammation in RA are complex since systemic inflammation may interfere with lipoprotein metabolism, leading to both qualitative and quantitative changes in triglycerides, HDL and LDL cholesterol fractions [7]. Thus, a lipid paradox is described in RA, with low levels of total and LDL cholesterol during the active phase of the disease, even though patients are at increased risk of CV events [8].

Due to the systemic inflammation and under the pressure of proinflammatory cytokines, a loss of lean mass has been described in RA as rheumatoid cachexia [9]. Conversely, RA is associated with other changes in body composition, with an excess of fat mass. Different phenotypes of body composition have been described in RA including overweight, sarcopenia, but also sarcopenic obesity [10]. The measurement of body composition in RA is currently feasible in routine practice using the reference technique, namely dual-energy X-ray absorptiometry (DEXA). An excess of fat mass has been reported in RA, with or without concomitant sarcopenia [10, 11]. Obesity may contribute to RA pathophysiology by secreting cytokines and specific adipose products, namely adipokines. Adipokines are predominantly secreted by the white adipose tissue and may represent a possible link between adiposity, RA, inflammation and metabolic complications such as metabolic syndrome and CV risk [12]. A wide range of adipokines are described and they all play physiological roles in various processes such as energy expenditure, appetite, coagulation and inflammation. For instance, leptin, resistin and visfatin have pro-inflammatory properties [13]. Adiponectin is an adipokine with metabolic functions implicated in insulin sensitivity and protection against diabetes and metabolic syndrome [14]. Adiponectin is considered to have both pro- and anti-inflammatory influences, depending on its isoforms [15]. Indeed, different isoforms of adiponectin are described, including low and high molecular weight (HMW) forms [16]. In addition, adiponectin, and especially its HMW isoform, is thought to have anti-atherogenic properties and a beneficial cardiometabolic profile [17].

Changes in lipoprotein profile have been reported in RA during randomized clinical trials evaluating the anti-IL-6 blocking agent tocilizumab (TCZ) [18]. Notably, a significant increase in total and LDL cholesterol levels after 8–12 weeks of treatment has been described, but without parallel changes in atherogenic index. A limited number of studies have evaluated the variation in serum adipokines (including adiponectin) during TCZ treatment [19–21]. In parallel, it is not well known whether TCZ may influence body composition and fat mass in RA. Thus, we conducted this study with the following aims: (i) to describe the changes in serum adipokines involved in inflammation and metabolic control, especially adiponectin and its HMW isoform; (ii) to evaluate changes in body composition, with a special focus on fat mass located in the android/abdominal region, a body area associated with cardiometabolic complications [22].

## Patients and methods

The ADIPRAT (*évolution des ADIpokines et de la composition corporelle chez les patients atteints de Polyarthrite Rhumatoïde et recevant un traitement par Tocilizumab*) study was a phase IV open-label multicentre study conducted in France (ClinicalTrials.gov: NCT02843789).

### Patients

Patients with RA defined according to the 2010 American College of Rheumatology (ACR) criteria, from 16 different rheumatology centres in France were enrolled between March 2013 and December 2016. Inclusion criteria were: (i) patient with active disease as defined by a disease activity score-erythrocyte sedimentation rate (DAS28-ESR) ≥ 3.2; (ii) disease not adequately controlled despite current treatment, including conventional synthetic disease-modifying anti-rheumatic drugs (csDMARDs) and/or biological DMARDs (bDMARDs); and (iii) the decision to start TCZ treatment was at the treating rheumatologist’s discretion and made jointly with the patient through shared decision-making. Exclusion criteria were previous exposure to TCZ or a contraindication to TCZ treatment.

#### Clinical assessments

At baseline, we recorded clinical parameters, namely socio-demographic data, comorbidities including metabolic syndrome (according to National Cholesterol Education Program [NCEP] Adult Treatment Panel III [ATP III] criteria) [23], past medical conditions, CV risk factors, smoking status, disease duration, extra-articular manifestations, current treatment for RA (csDMARDs) including the use of glucocorticoids (GC) and previous bDMARDs. Patients were evaluated at baseline (month 0 [M0]), and then at months (M) 1, 3, 6 and 12. At each time point, parameters that were collected included weight, height, body mass index (BMI) (weight/height^2^), waist circumference (measured at the top of the iliac crest), waist-to-hip ratio (ratio of waist to hip circumference; hip circumference was measured at the widest portion of the buttock), DAS28-ESR and health assessment questionnaire (HAQ) score. The different outcome measures were recorded by the same rheumatologist in each centre.

#### Tocilizumab treatment

Patients received TCZ IV 8 mg/kg monthly as administered in daily practice. The duration of the study was 12 months and the rheumatologist was free to reduce TCZ dosage to 4 mg/kg at his/her discretion, as required. Tocilizumab could be administered as monotherapy or in combination with a csDMARD. During follow-up, GC could be reduced, if applicable.

Written informed consent was obtained from each participant and this study was approved by the local ethics committee (*Comité de Protection des Personnes CPP Est-II*, reference number: 11/616).

## Methods

### Laboratory assessments

Blood samples were obtained from each patient at each time point, in the morning (8.00 AM) after an overnight fast. The blood samples (without anticoagulant) were immediately centrifuged (for 10 min at 1500*g*) and serum was stored at − 80 °C until analysis. Routine laboratory variables that were analysed in each centre the day of the visit and without previous freezing included complete blood cell count, serum creatinine, transaminases, inflammatory parameters (ESR, CRP), glycaemia and lipids (total cholesterol, LDL, HDL cholesterol and triglycerides). The atherogenic index was calculated as the ratio of total/HDL cholesterol. Rheumatoid factors and anti-cyclic citrullinated peptides (CCP) antibodies were determined at baseline if unknown. Analyses that were performed on frozen serum samples were circulating IL-6, serum adipokines, insulinaemia and serum ghrelin, a gastric peptide involved in appetite regulation. These analyses were all analysed in a central laboratory (Medical biochemical laboratory, University Hospital of Besancon, France). Total adiponectin, total ghrelin, leptin and insulin were determined by radioimmunoassay (RIA) (Merck Millipore Corp, Billerica, MA, USA). The inter-assay coefficients of variation were 9.3, 16, 3.6 and 3.8% for total adiponectin, total ghrelin, leptin and insulin, respectively. Resistin and HMW adiponectin levels (R&D systems Europe Ltd., Lille, France) were measured by quantitative sandwich enzyme-linked immunosorbent assay (ELISA) and inter-assay coefficients of variation were 7.8 and 8.5%, respectively. The lowest level that could be detected by each assay (sensitivity) was 0.44 ng/mL for leptin, 1 μg/mL for total adiponectin, 1 μg/mL for HMW adiponectin, 0.06 ng/mL for resistin, 93 pg/mL for total ghrelin and 2.7 μU/mL for insulin.

The homeostasis model assessment for insulin resistance (HOMA-IR), calculated as fasting insulin (μU/mL) × fasting glucose (mmol/L)/22.5, was used to estimate insulin resistance [24]. Since leptin levels are strongly associated with the amount of adipose tissue, we determined leptin corrected for fat mass (leptin/fat mass).

### Measurements of body composition

A total body scan was performed using a Lunar densitometer (iDXA or Prodixy; GE Healthcare, Madison, WI, USA). Measurements were performed at baseline and then at M6 and M12. Subjects were scanned using standard imaging and positioning protocols according to the manufacturer’s instructions. Body composition was studied from the total body scan, with measurements of fat mass and lean mass. Total and regional body fat mass and lean mass were determined. Adiposity (% fat) was defined as the ratio of total fat tissue to (total lean mass + total fat tissue). Fat distribution was evaluated as the relative proportion of fat tissue in the android (abdominal) and gynoid (hip and thigh) regions. For the measurement of android fat, a region of interest was automatically defined (from the top of the iliac crest to 20% of the distance from the top of the iliac crest to the base of the skull). In parallel to body composition assessment, bone measurements at the lumbar spine (LS) and femoral neck (FN) were recorded, including bone mineral density (BMD) measurements at the lumbar spine (L1 to L4, antero-posterior view), the left and right femoral necks and the total skeleton. The results were given as *T* score. Quality control scans and calibration were performed daily in each centre during the study period by using the manufacturer’s standards.

#### Statistical analysis

Our main outcome measure was the change in adiponectin (total and HMW adiponectin) at month 6. The number of subjects was calculated using the following assumptions*:* (i) 20% change in adiponectin after 6 months of treatment (13.2 to 10.6 μg/mL), (ii) standard deviation of 4.3, (iii) alpha risk of 5% and a power of 90% and (iv) bilateral situation, based on changes in adiponectin that were previously reported during TNF alpha inhibitor (TNFi) treatment in RA [25]. Results are expressed as mean ± standard deviation (SD) for quantitative variables, and as number and percentage for categorical variables. Figures are given with mean ± standard error of mean (SEM). Results were obtained at each time point (M0 to M12) and quantitative variables were compared between M0 and each subsequent time point using the paired Student *t* test. Due to multiple comparisons for total and HMW adiponectin (*N* = 4), a Bonferroni correction was used, resulting in a significant level of 0.0125. Sensitivity analysis was performed in order to analyse the impact of missing data on the results using an expectation-maximization algorithm (PROC MI - SAS). Fisher’s exact test was used for comparing the response to TCZ according to smoking habit. According to the low number of patients in specific patient subgroups and the non-normality of the distribution (using Shapiro-Wilk test), the variation of total and HMW adiponectin according to EULAR response was evaluated by Kruskall-Wallis test and Dwass-Steel-Crichtlow-Fligner test for post hoc analysis. The relationships between the changes in serum adipokines and the changes in laboratory parameters of inflammation (ESR, CRP, IL-6) or disease activity (DAS28-ESR) at M6 and at M12 were evaluated by the goodness-of-fit (*R*^2^) from linear regression. The same test was used to analyse the correlation between the changes in lean mass and the changes in laboratory parameters of inflammation or disease activity (DAS28-ESR) at M12. All statistical analyses were performed by the biostatistics unit of the University Hospital of Besancon (uMETh, M. Puyraveau, biostatistician) using SAS v9.4 (SAS Institute Inc., Cary, NC, USA).

## Results

### Baseline characteristics of the patients

**Fig. 1:**
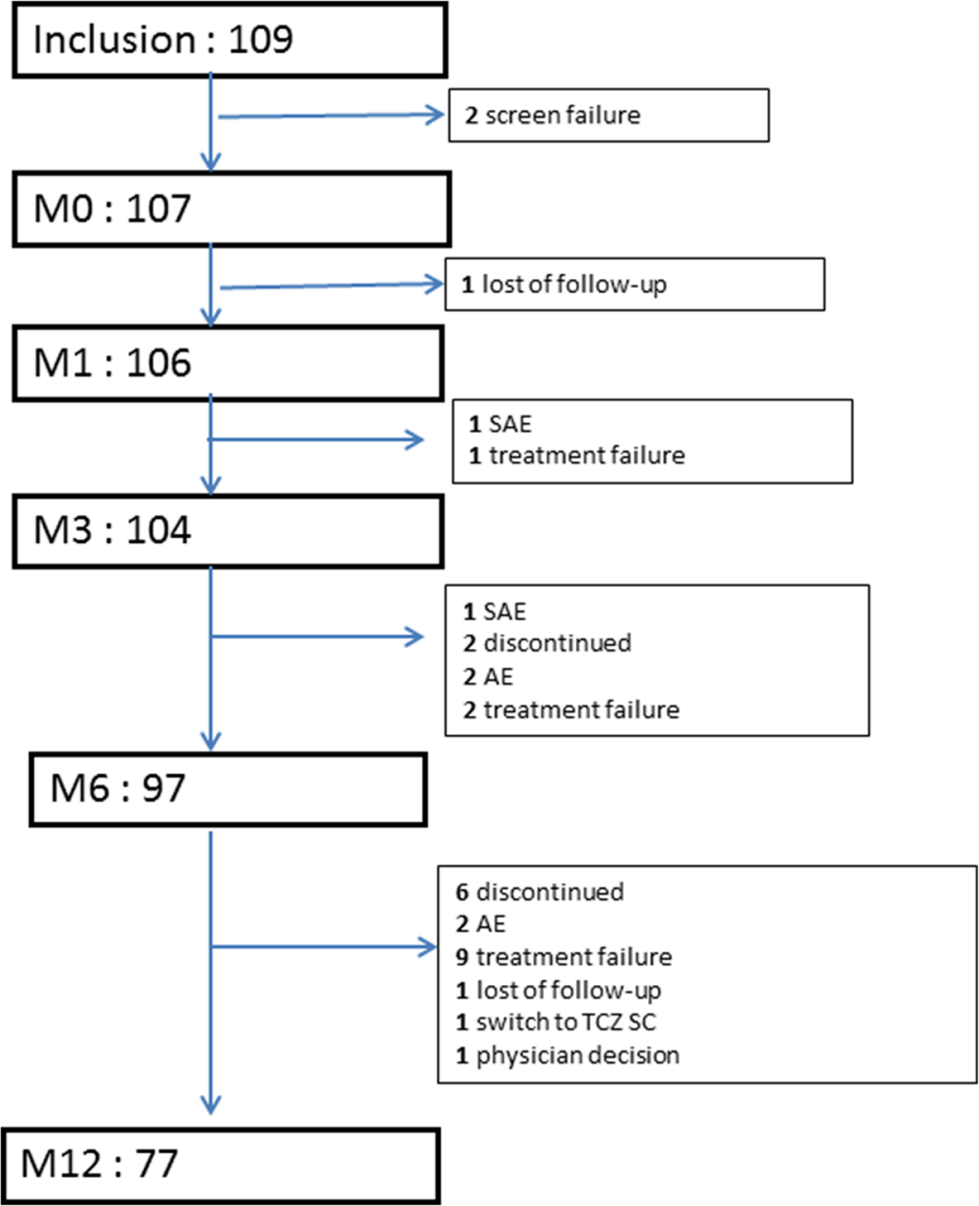
Flow chart of the study. M, month; SAE, serious adverse event; AE, adverse event; TCZ SC, tocilizumab subcutaneous

Figure 1 summarizes the flow chart of the study. Among the 109 patients who were eligible for the study, 2 were not included due to screening failure. Accordingly, 107 patients were thus included. One patient was lost to follow-up after M0, 2 withdrew after M1, 7 after M3 and 20 after M6. The reasons for study discontinuation are detailed in Fig. 1. A total of 77 patients were still on treatment at the end of the study. The demographics and clinical variables of the study population at baseline are shown in Table 1. Patients all had active disease as defined by DAS-28 ESR. Most of the patients were women, and most had positive rheumatoid factors and/or anti-CCP antibodies. A small proportion of patients had CV risk factors. A high proportion of patients had erosive disease. Most had received previous bDMARD treatment (Table 1). Tocilizumab was given IV at 8 mg/kg for almost all patients during the study (100% at M0 and M1, 93.3% at M3, 93.8% at M6 and 98.7% at M12), and only a small proportion received TCZ 4 mg/kg according to the treating rheumatologist’s decision. The treatment was given in combination with a csDMARD for the majority of patients, mainly methotrexate (MTX) or alternatively, leflunomide (LEF) (Table 1). Among the patients included, none had metabolic syndrome.

**Table 1:** Clinical and biological characteristics of the study population at baseline

Characteristics	***N*** = 107
Age (years)	56.6 ± 13.5
Disease duration (years)	9.9 ± 8.1
Gender (M/F)	29 (27.1)/78 (72.9)
DAS28-ESR	4.93 ± 1.3
Extra articular manifestations	
Nodules	18 (16.8)
Sicca syndrome	15 (14)
Vasculitis	2 (1.8)
X-ray erosions	
Presence	68 (66.6)
Absence	34 (33.3)
ND	5 (4.6)
csDMARDs	77 (71.9)
MTX	66 (61.7)
LEF	10 (9.3)
SLZ	0
HCQ	1 (0.9)
Glucocorticoids	74 (69.2)
Dosage (mg/day)	6.8
Previous bDMARDs	69 (64.5)
TNFi	67 (62.6)
Rituximab	5 (4.6)
Abatacept	13 (12.1)
Anakinra	1 (0.9)
Rheumatoid factors	81 (75.7)
Anti-CCP antibodies	82 (76.6)
CV risk factor:	
Hypertension	40 (37.3)
Diabetes^#^	7 (6.5)
Dyslipidaemia^##^	19 (17.7)
Smoking	
Never	65 (60.1)
Previous	20 (18.7)
Current	22 (20.5)

Results are given as mean ± SD or *N* (%)

*M* male, *F* female, *csDMARDs* conventional synthetic disease-modifying anti-rheumatic drugs, *bDMARDs* biological disease-modifying anti-rheumatic drugs, *TNFi* TNFalpha inhibitor, *MTX* methotrexate, *LEF* leflunomide, *SLZ* sulfasalazine, *HCQ* hydroxychloroquine, *ND* not determined

^#^Diabetes treatments: insulin *N* = 2, metformin *N* = 6, repaglinide *N* = 2, liraglutide *N* = 1, glicazide *N* = 1, sitagliptin *N* = 1

^##^Dyslipidaemia treatments: statins *N* = 14, ezetimibe *N* = 3, fibrates *N* = 2

### Effects of TCZ on disease activity and inflammatory parameters

There was a significant improvement in disease activity as shown by a decline in DAS28-ESR (DAS28-ESR M0 vs M6, *p* <  10^−4^; M0 vs M12, *p* <  10^−4^; analysis with missing data, *p* <  10^−4^) (Table 2). At the end of the study (M12), among the 77 patients still receiving the treatment, 64.5 and 17.1% of patients achieved remission or low disease activity, respectively, according to the EULAR definition. Response to TCZ treatment at M12 was also analysed according to smoking status: the percentage of patients in remission did not differ between the current and the previous/never smokers (64.3% vs 64.5%); the proportion of patients in low disease activity was higher in previous/never smokers compared to current smokers, but results were not significant (*p* = 0.39). For the whole group of patients, the percentage of non-responders, moderate and good responders according to the EULAR definition was 19,16.8 and 64.2% at M6 and 6.6, 21 and 72.4% at M12, respectively. In parallel, HAQ score significantly decreased over the study period (Table 2). Similar results were observed for laboratory parameters of inflammation, ESR and CRP levels. Circulating IL-6 increased under TCZ treatment, a result that was only significant at M6 (Table 2). There were no changes in concomitant medication by csDMARDs during the 12-month follow-up while GC were reduced from 6.8 mg/day at baseline to 5.1 mg and 4.2 mg at M6 and M12, respectively.

**Table 2:** Disease activity, HAQ score and laboratory parameters of inflammation during the study

	M0 (***N*** = 107)	M1 (***N*** = 106)	M3 (***N*** = 104)	M6 (***N*** = 97)	M12 (***N*** = 77)	P*, M0 vs M6 (***N*** = 97)	P*, M0 vs M12 (***N*** = 77)	***P***^**#**^
**DAS28-ESR**	4.93 ± 1.3	3.55 ± 1.2	2.8 ± 1.2	2.5 ± 1.2	2.3 ± 1.3	**< 10**^**−4**^	**< 10**^**−4**^	**< 10**^**−4**^
**HAQ**	1.4 ± 0.6	Not evaluated	Not evaluated	0.97 ± 0.6	0.98 ± 0.6	**< 10**^**−4**^	**< 10**^**−4**^	**< 10**^**−4**^
**ESR (mm/h)**	27.8 ± 22.8	8.2 ± 8.4	6.5 ± 10.2	6.1 ± 7.1	5.8 ± 1.8	**< 10**^**−4**^	**< 10**^**−4**^	**< 10**^**−4**^
**CRP (mg/L)**	17.7 ± 26.7	5.4 ± 15.4	4.7 ± 12.8	2.4 ± 4.4	2.3 ± 4.5	**< 10**^**−4**^	**< 10**^**−4**^	**< 10**^**−4**^
**IL-6 (pg/mL)**	26.4 ± 37.8	72.3 ± 76	62.3 ± 78.7	53.1 ± 58.7	53.8 ± 110	**< 10**^**−4**^	0.04	0.04

Results are given as mean ± SD

*M* month, *HAQ* health assessment questionnaire, *ESR* eythrocyte sedimentation rate, *CRP* C-reactive protein

*Paired Student’s *t* test

^#^Sensitivity analysis

### Effects of TCZ on adiponectin and other adipokines and on metabolic parameters

**Fig. 2:**
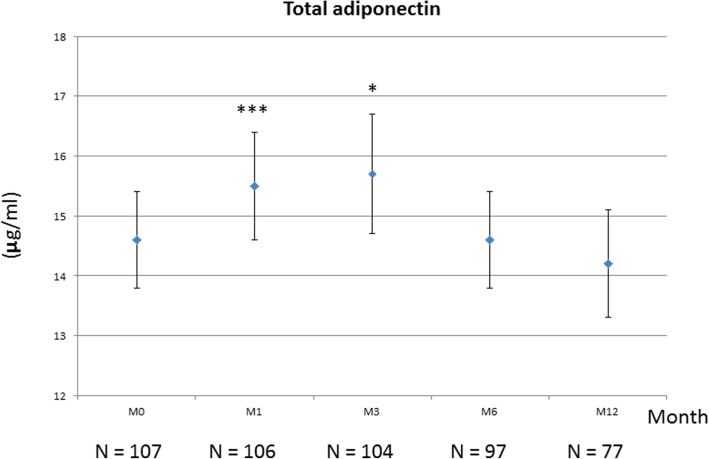
Changes in total adiponectin during tocilizumab treatment. Patients received IV tocilizumab monthly for 12 months with or without csDMARDs. They were evaluated at month M0, M1, M3, M6 and M12. The number of patients at each visit is indicated. Results are shown as mean ± SEM (paired Student’s *t* test: ***p = 0.0022 at month 1; *p = 0.0105 at month 3)

**Fig. 3:**
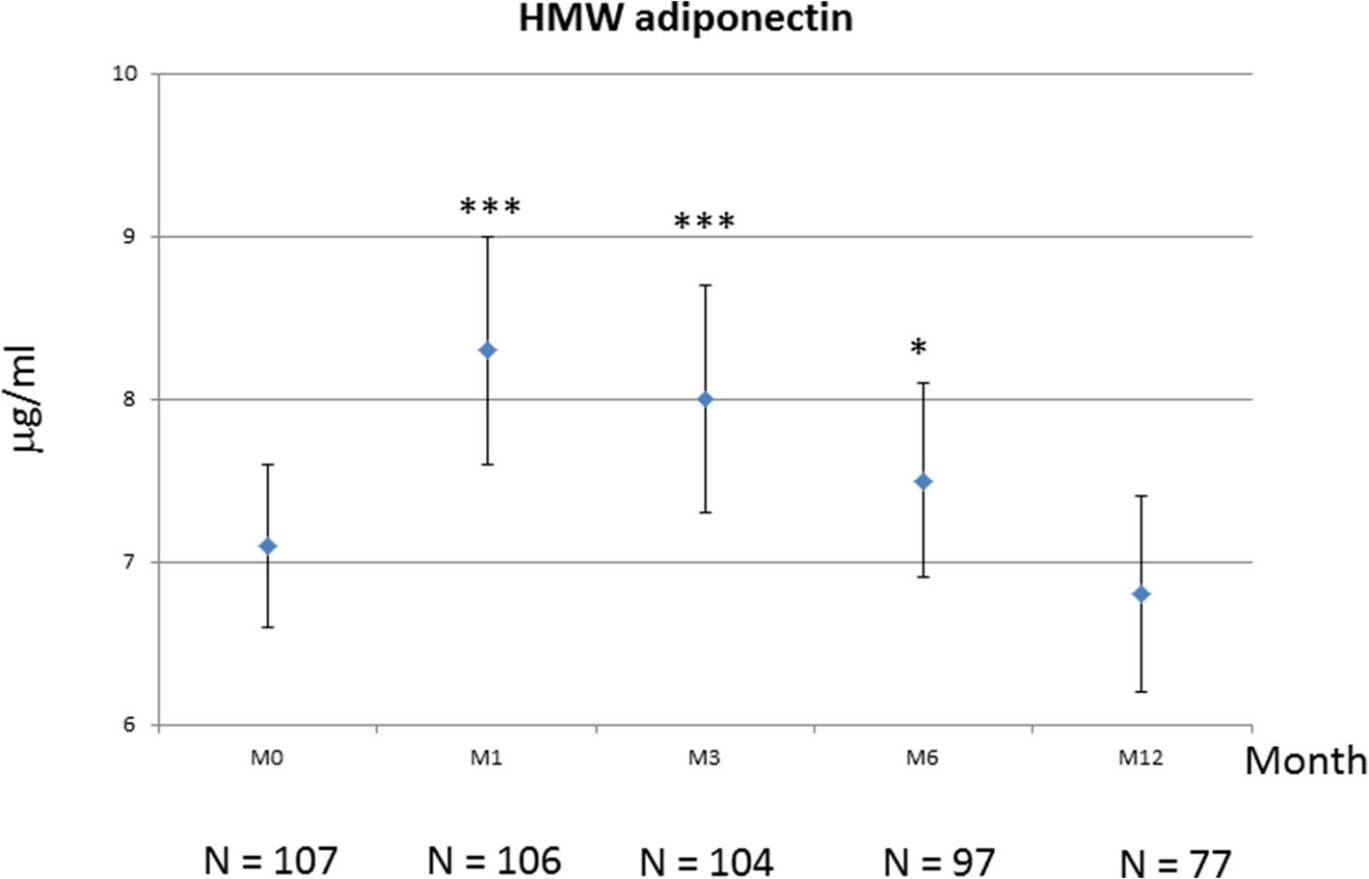
Changes in high molecular weight (HMW) adiponectin during tocilizumab treatment. Patients received IV tocilizumab monthly for 12 months with or without csDMARDs. They were evaluated at month M0, M1, M3, M6 and M12. The number of patients at each visit is indicated. Results are shown as mean ± SEM (paired Student’s *t* test: ***p < 0.0001 at month 1 and *p* = 0.0018 at month 3; *p = 0.011 at month 6)

Total adiponectin increased between M0 and M3 (+ 8%), with a difference that was significant at M1 (*p* = 0.0022) and M3 (*p* = 0.0105) and but not at M6 (*p* = 0.055) (Fig. 2 and Table 3). Indeed, serum total adiponectin reached at peak at M3, then progressively declined to return to baseline values (Fig. 2). Changes between M0 and M12 were not significant, even after imputation analysis. The variation in HMW adiponectin was similar, but more rapid, with a significant peak observed as early as M1 (+ 13.7%)(*p* < 0.0001), which persisted at M3 (*p* = 0.0018) (Fig. 3), and then declined at M6 (*p* = 0.011) to return progressively to baseline values at M12, with a difference between M0 and M12 that was not significant (Table 3 and Fig. 3). Conversely, imputation analysis taking account missing data showed a significant variation of HMW adiponectin between baseline and M12 (*p* = 0.01) (Table 3). When examining the changes of adiponectin (total and HMW) in patients with concomitant csDMARDs, these variations were not significant as well as were the changes in patients without concomitant csDMARDs (Table 3). The changes in total and HMW adiponectin between baseline and each time point were then examined according to EULAR response, i.e. between the non-responders, moderate and good responders to TCZ. Except for total adiponectin at M1 (*p* = 0.012), these changes did not differ between the 3 groups of patients (all *p* > 0.05) (Table 4). For the other adipokines (leptin, resistin) and ghrelin, no significant variation was observed (Table 3). In parallel, there was no significant variation in glycaemia, insulinaemia and HOMA-IR over the study period. Total cholesterol and LDL cholesterol significantly increased at M6, while HDL cholesterol, triglycerides and atherogenic index were unchanged compared to pre-TCZ values. LDL cholesterol elevation persisted at M12 (Additional file 1). Treatments for diabetes and dyslipidaemia were stable over the study period for all patients.

**Table 3:** Changes in serum adipokines during the study

	M0 (***N*** = 107)	M1 (***N*** = 106)	M3 (***N*** = 104)	M6 (***N*** = 97)	M12 (***N*** = 77)	P*, M0 vs M6 (***N*** = 97)	P*, M0 vs M12 (***N*** = 77)	***P***^**#**^
**Total adiponectin (μg/mL)**	14.54 ± 8.1	15.5 ± 8.7	15.7 ± 9.9	14.6 ± 7.5	14.2 ± 7.6	0.055	0.25	0.3
**Total adiponectin (**μg**/mL), TCZ alone**	15.18 ± 7.8	14.75 ± 7.9	14.76 ± 6.6	15.03 ± 7.5	14.23 ± 6.6	0.34	0.8	0.31
**Total Adiponectin (**μg**/mL), TCZ and csDMARDs**	14.40 ± 8.3	15.82 ± 9.1	16.04 ± 11.0	14.47 ± 7.6	14.14 ± 8.2	0.1	0.24	0.019
**HMW adiponectin (μg/mL)**	7.3 ± 5.4	8.3 ± 6.6	8 ± 6.4	7.5 ± 5,4	6.8 ± 4.6	**0.011**	0.057	**0.01**
**HMW adiponectin (μg/mL), TCZ alone**	7.32 ± 4.50	7.87 ± 5.40	7.94 ± 5.22	7.97 ± 5.21	7.03 ± 4.72	0.052	0.046	0.09
**HMW adiponectin (μg/mL), TCZ and csDMARDs**	7.07 ± 5.28	8.42 ± 7.08	8.06 ± 6.84	7.32 ± 5.47	6.73 ± 4.66	0.045	0.3	0.045
**Leptin (ng/mL)**	32.64 ± 26.9	32.45 ± 25.4	31.7 ± 23.6	32.6 ± 27.3	32.8 ± 27.5	0.059	0.17	0.22
**Leptin/fat mass**	1.08 ± 0.7	ND	ND	1.1 ± 0.8	1.2 ± 1.4	0.049	0.2	0.14
**Resistin (ng/mL)**	10.5 ± 4.9	11 ± 4.9	10.9 ± 4.6	10.8 ± 4,9	10.9 ± 5.3	0.53	0.16	0.13
**Ghrelin (pg/mL)**	2121.9 ± 1309.4	2031.5 ± 1266	2072.9 ± 1299	2074.8 ± 1278.4	2035 ± 1304.3	0.78	0.19	0.28

Results are given as mean ± SD

*M* month, *TCZ* tocilizumab, *HMW* high molecular weight, *ND* not determined

*Paired Student’s *t* test

^#^Sensitivity analysis

**Table 4:** Comparison of changes in total and HMW adiponectin between non-responders, moderate responders and good responders (EULAR definition) at each time point (results are given as mean ± SD; *N* number of subjects; *P*^#^: Kruskal Wallis test. Results of post hoc analysis [Dwass-Steel-Crichtlow-Fligner test] for total adiponectin at M1: moderate versus good responders: *p* = 0.0482; non-responders versus good responders: *p* = 0.974)

	Adiponectin (훍g/mL)	Non-responders	Moderate responders	Good responders	***P***^**#**^
M1	Total	0.28 ± 3.4 (28)	2.41 ± 3.2 (41)	0.15 ± 3.8 (24)	**0.012**
	HMW	0.53 ± 1.6 (27)	1.49 ± 3 (41)	0.82 ± 1.7 (24)	0.6
M3	Total	1.55 ± 3.4 (15)	1.9 ± 5.7 (31)	0.78 ± 4.3 (44)	0.9
	HMW	1.55 ± 3.4 (15)	1.9 ± 5.7 (31)	0.78 ± 4.3 (44)	0.9
M6	Total	− 0.87 ± 2.9 (17)	1.9 ± 3.3 (15)	1.17 ± 4.7 (52	0.078
	HMW	− 0.87 ± 2.9 (17)	1.9 ± 3.3 (15)	1.17 ± 4.7 (52	0.078
M12	Total	3.1 ± 6.9 (4)	- 0.06 ± 4.5 (14)	0.77 ± 5.1 (46)	0.48
	HMW	0.78 ± 1.9 (4)	- 0.05 ± 2.1 (14)	0.53 ± 1.6 (47)	0.42

### Anthropometric measurements and body composition changes under TCZ treatment

Mean weight and BMI increased significantly, a change that was observed as early as M3, and remained significant at M6 and M12 as compared to baseline (Table 5). Waist circumference also significantly increased but remained stable after M6. Conversely for waist-to-hip ratio, there was no significant variation. Body composition measurements by DEXA showed that there was a continuous gain in lean mass over the course of study, with a significant change at M6. Conversely, fat mass and % fat were not modified by the treatment, nor was fat in the android and gynoid regions (Table 5).

**Table 5:** Anthropometric measurements and body composition changes during the study

	Baseline (***N*** = 107)	M1 (***N*** = 106)	M3 (***N*** = 104)	M6 (***N*** = 97)	M12 (***N*** = 77)	P*, M0 vs M6 (***N*** = 97)	P*, M0 vs M12 (***N*** = 77)	***P***^**#**^
**Weight (kg)**	70.3 ± 15.1	70.3 ± 14.8	71 ± 15.7	70.9 ± 15.3	72 ± 15.3	**0.0037**	**0.0003**	**0.0005**
**Body mass index (kg/m**^**2**^**)**	26.4 ± 5.5	26.4 ± 5.3	26.7 ± 5.7	26.7 ± 5.5	27.2 ± 5.8	**0.0165**	**0.0006**	**0.0006**
**Waist circumference (cm)**	90.3 ± 13.2	90.9 ± 15.5	93.4 ± 17	93.2 ± 14.7	93.9 ± 15.3	**0.0008**	**0.006**	**0.0002**
**Waist-to-hip ratio**	0.95 ± 0.06	0.95 ± 0.06	0.93 ± 0.06	0.95 ± 0.12	0.94 ± 0.1	0.94	0.77	0.9
**Lean mass (kg)**	40.76 ± 8.4	Not evaluated	Not evaluated	41.79 ± 8.8	42.11 ± 8.9	**0.0097**	0.021	0.028
**Fat mass (kg)**	27.7 ± 12.1	Not evaluated	Not evaluated	27.4 ± 10.7	28.15 ± 11.4	0.69	0.88	0.8
**% fat**	38.9 ± 10.3	Not evaluated	Not evaluated	38.7 ± 9.1	39.3 ± 9,8	0.59	0.56	0.6
**Fat mass android region (kg)**	2.52 ± 1.3	Not evaluated	Not evaluated	2.51 ± 1.3	2.67 ± 1.5	0.99	0.51	0.25
**Fat mass gynoid region (kg)**	4.5 ± 1.9	Not evaluated	Not evaluated	4.7 ± 2.7	4.6 ± 1.9	0.36	0.55	0.19

Results are given as mean ± SD

*M* month

*Paired Student’s *t* test

^#^Sensitivity analysis

### Correlation analysis between changes in serum adiponectin, lean mass and changes in disease activity

Since significant changes were observed for adiponectin (total and HMW adiponectin), we next analysed the relationships between the changes in serum levels of this adipokine and the changes in clinical and laboratory parameters of disease activity at M6 and M12. There was a strong correlation between the variation in both total adiponectin and HMW adiponectin, and ESR, CRP, IL-6 and DAS28-ESR at month 6 (*R*^2^ between 0.76 and 0.99) while similar correlations were observed with ESR, CRP and IL-6 at month 12 (*R*^2^ between 0.64 and 0.94), but not with DAS28-ESR (Table 6). No such relationships were observed for the other adipokines. Finally, variation in lean mass was strongly correlated with changes in laboratory and clinical parameters of disease activity (ESR, CRP, IL-6 and DAS28-ESR) at M12 (*R*^2^ coefficients: 0.96, 0.97, 0.89 and 0.96, respectively).

**Table 6:** Relationships between changes in serum adipokines and changes in laboratory parameters of inflammation or disease activity at months 6 and 12 (*R*^2^: goodness-of-fit from linear regression)

*R*^2^	Time of assessment	ESR	CRP	IL-6	DAS28-ESR
Total adiponectin	M6	0.99	0.98	0.89	0.93
	M12	0.78	0.75	0.88	0.64
HMW adiponectin	M6	0.92	0.93	0.99	0.76
	M12	0.67	0.64	0.94	0.42
Leptin	M6	0.37	0.46	0.09	0.53
	M12	0.81	0.81	0.36	0.94
leptin/fat mass	M6				
	M12	0.91	0.45	0.96	0.91
Resistin	M6	0.19	0.11	0.51	0.08
	M12	0.26	0.31	0.27	0.51
Ghrelin	M6	0.32	0.31	0.74	0.01
	M12	0.09	0.04	0.23	0.07

*HMW* high molecular weight

BMD measurements (LS, FN and total skeleton) were also analysed but results (data not shown) showed no significant variation for BMD or T score excepting FN *T* score with a significant decrease at M12 (M0 vs M12: T score − 0.89 ± 1.1 vs − 1.04 ± 1.1, *p* = 0.0009).

## Discussion

Our results show that in patients with active RA despite csDMARDs and/or bDMARDs, IV TCZ, in combination with MTX (or other csDMARDs) or as monotherapy, may modulate serum levels of adiponectin, notably both the total and HMW isoforms.

Adiponectin is a collagen-like protein mainly produced by adipocytes, and it has predominantly metabolic functions [14, 26]. It is a multimeric protein that exists in different isoforms, each with distinct biological properties. Different isoforms are described, including globular adiponectin and a full-length form, their assemblage leading to monomeric subunits. Monomeric adiponectin can trimerize resulting in low molecular weight (LMW) adiponectin. This isoform can assemble to form a HMW isoform [14, 16]. There is an accumulating body of evidence demonstrating that hypoadiponectinaemia is associated with the development of insulin resistance, type 2 diabetes and metabolic syndrome and that adiponectin may be considered as a surrogate marker for atherosclerosis and CV risk [27, 28]. Indeed, hypoadiponectinaemia is a strong risk factor for CV disease [27]. The mechanisms that may explain such favourable cardiometabolic effects include multiple potent anti-atherogenic properties such as beneficial effects on endothelial cells as well as improvement of endothelial dysfunction [28]. Adiponectin is thus viewed as a protective factor for CV disease and as a defensive adipokine [27]. However, in RA, the relationships between adiponectin and its HMW isoform with cardiometabolic risk and/or surrogate markers of atherosclerosis appear less evident [29]. In parallel, adiponectin, as well as other adipokines, can interact with the immune system by modulating the immune response. Contrary to leptin or resistin, it is established that adiponectin has anti-inflammatory properties by inhibiting the production of proinflammatory cytokines and expression of adhesion molecules, or by stimulating the release of anti-inflammatory factors [30]. Conversely, experimental data suggest that adiponectin may have pro-inflammatory effects by stimulating inflammatory cytokine production and metalloprotease release, by promoting angiogenesis and finally by stimulating immune cells to produce inflammatory mediators [15]. However, adiponectin must be viewed as a molecule with a dual function in inflammation, displaying both pro- and anti-inflammatory properties. This dual characteristic is related to its different isoforms. Indeed, it is considered that LMW isoforms have anti-inflammatory effects, while the opposite is the case for the HMW isoforms [16]. In addition, the cardiometabolic protective effects of adiponectin have been more closely related to the HMW isoform than total adiponectin in diabetic subjects [31].

Adiponectin is a relevant adipokine for the pathophysiology of RA. Indeed, serum adiponectin has been found to be increased in RA patients compared to control subjects and can be detected in the synovial fluid of patients [16]. Some but not all studies found that total adiponectin correlated with disease activity and it has been reported that adiponectin may be associated with structural damage [32] and radiographic progression [33, 34]. Limited data exist on the changes in adiponectin during treatment with bDMARDs in RA. In patients receiving TNFi, we and others did not observe significant changes in total adiponectin, while HMW adiponectin decreased during 2 years of administration of TNFi in patients with RA or ankylosing spondylitis (AS) [35]. Changes in total adiponectin under TCZ have been evaluated in 3 previous studies. Total adiponectin was found to increase after 3 months of TCZ administration in 11 non-diabetic patients with RA [19]. In a second study evaluating serum adipokines in a limited number of patients receiving TCZ, serum total adiponectin was not modified after 6 months [20]. In a 6-month follow-up study involving 44 patients with RA who received IV TCZ 8 mg/kg, a significant increase in total adiponectin was observed [21]. These changes were described in patients with or without concomitant MTX in that study. These results are in keeping with ours, showing that total and HMW adiponectin significantly increased during the first 3 months of treatment. These significant changes of total and HMW adiponectin were not observed when we examined separately patients receiving concomitant csDMARDs and those without csDMARDs. This may probably be explained by the small number of patients in these subgroups. HMW adiponectin was not evaluated in the three previous studies [19–21]. Our results showed an upregulation of adiponectin, both total and HMW, especially during the first 3 months of treatment, and this effect was attenuated afterwards. The change in HMW between baseline and month 6, albeit significant, was low (difference of 0.2 μg/mL) raising the question of its biological relevance. Alternatively, the changes of both total and HMW adiponectin during the first months of TCZ administration were more evident, and this may have biological consequences.

We did not observe changes in serum leptin or resistin in our study. Leptin is another adipokine involved in appetite regulation with prominent pro-inflammatory effects [13]. TNFi administration in RA has various effects on serum leptin, notably decreasing serum levels [35]. TCZ was shown to decrease serum leptin at 6 months in one study [20] but results were not corrected for fat mass. We did not observe changes in serum leptin in our series, while leptin/fat mass ratio marginally increased at 6 months of treatment. Resistin is another adipokine involved in insulin resistance and type 2 diabetes and, in parallel, has pro-inflammatory properties [13]. TNFi may reduce serum levels of resistin in patients with RA or AS [35], but TCZ did not seem to have a similar influence, as suggested by our results and those reported by other groups [20, 21]. The relationships between pro-inflammatory cytokines such as TNFα or IL-6 and adiponectin production are not well understood. TNFα is considered to be a strong inhibitor of adiponectin promoter activity [26]. IL-6 and TNFα secreted by adipose tissue may inhibit the local production of adiponectin [36]. IL-6 is able to reduce adiponectin release in combination with exogenous soluble IL-6 receptors from human adipocytes [27], an effect that is in keeping with our results. The elevation in serum adiponectin and its HMW isoform was mainly observed during the first months of TCZ administration in our study. We can highlight from these results that circulating IL-6 temporarily increases following TCZ administration, an effect that culminates after 1 month of treatment, with a secondary decrease to levels higher than baseline values [37]. Similar effects were described for soluble IL-6 receptors with an increase as early as 1 month of treatment, then reaching a plateau [37]. In addition, we observed close relationships between the changes in total and HMW adiponectin on the one hand, and the changes in parameters of disease activity (ESR, CRP, DAS28-ESR and also IL-6) on the other hand. These parallel changes suggest that controlling inflammation by IL-6 blockade may induce the release of total and HMW adiponectin.

We also evaluated body composition during TCZ treatment in the present study. In RA, previous studies have reported an excess of visceral adipose tissue located in the abdominal/visceral region, especially in women [38]. Changes in body composition, especially adipose tissue, during bDMARD treatment have been examined in a limited number of studies. During TNFi administration, a gain of weight due to fat mass increase has been reported and the fat is distributed to the abdominal/visceral region, raising the issue of its impact on CV risk [35]. The influence of TCZ treatment on body composition has rarely been examined [20]. Our results showed no changes in fat mass or adiposity, whereas there was a weight gain due to lean mass increase, especially at 6 months. Concomitantly, waist circumference increased but without a parallel change in waist-to-hip ratio, a strong indicator of visceral adiposity and predictor of CV disease. However, waist circumference was enhanced during the first 3 months of treatment, then remaining stable, while BMI and lean mass increased gradually during the study. Visceral adipose tissue was not examined in our study due to the stability of fat mass in the android region. Our results are in line with those of a similar study in a series of 21 patients with RA receiving TCZ for 12 months [20]. In that study, lean mass, fat-free mass index but also appendicular lean mass and skeletal muscle mass index increased during the follow-up. In parallel, no change was observed for fat mass, including measurements on visceral adipose tissue. It was concluded that IL-6 inhibition had a favourable impact on skeletal muscle mass and may counteract the sarcopenic process of RA. Rheumatoid cachexia has been related to disease activity, and the production of pro-inflammatory cytokines, including TNFα but IL-6, is thought to be another contributing factor [9]. Disability secondary to functional impairment and GC use are additional factors that may explain a state of cachexia in RA. Our results are in keeping with those previously reported [20], confirming that IL-6 blockade is associated with a gain of lean mass in this large series of patients. The decrease in GC dosage may also partly explain these results, as may the improvement in physical function, as suggested by the improvement in the HAQ score. However, specific physical activity measurements were not performed in our study. How IL-6 inhibition may impact the lean/muscle mass is unknown, but probably results from anabolic effects on myocytes. Indeed, circulating IL-6 has established effects on skeletal muscle mass and metabolism [39]. IL-6, in conjunction with TNFα, may promote muscle loss and is associated with a decline in muscle mass and muscle strength, especially in older people. Indeed, elevated serum IL-6 has been associated with frailty and physical function in the ageing population [40]. In addition, muscle atrophy has been observed in IL-6 transgenic mice and this was reversed by IL-6 receptor blockade [41]. Thus, blocking the biological functions of IL-6 may positively impact on muscle mass, strength and physical function, a result that may explain the improvement in lean mass and HAQ score in our series. Whether IL-6 blockade may interfere with specific growth factors such as IGF-I and/or its binding proteins is currently unknown.

As expected and as previously reported [18, 42], lipid parameters increased during TCZ treatment, especially total and LDL cholesterol without alteration of atherogenic index. However, these changes are complex and inflammation itself has a wide range of consequences on lipids, lipoproteins and associated molecules as exemplified by the lipid paradox [7, 8]. Additional studies on lipid parameters during TCZ treatment in RA suggested valuable results with a reduction of phospholipase A2, HDL associated with serum amyloid A protein and also lipoprotein(a), all of which have pro-atherogenic properties [43]. The risk of major CV events during TCZ treatment was evaluated in a post hoc analysis of randomized clinical trials and extension studies. At baseline, total/LDL cholesterol ratio was independently associated with major CV events, but the risk during TCZ treatment was related to control of disease activity and not to lipid changes [44]. Finally, our results showed an elevation of adiponectin and its HMW fraction together with stability of fat mass, especially in the android/abdominal region, as well as a gain in lean/muscle mass, thus providing additional evidence of a favourable cardiometabolic profile of TCZ in RA. Such effects have not been described during TNFi treatment [35].

The present study has a number of limitations. This was an open-label, observational study without a control group. We were not able to demonstrate that our results were related to a direct or indirect effect of TCZ by controlling inflammation. Due to the real-life context, a number of patients discontinued the study, leading to missing data. Conversely, we performed our study in a large population of patients. Missing data were taken into account by appropriate analysis. They had minor impact on the results, and we were able to show significant changes in the primary parameter, adiponectin. Concomitant medication by MTX did not seem to influence adiponectin changes under TCZ treatment, as previously reported [21].

## Conclusion

TCZ treatment was associated with a significant increase in adiponectin, particularly its HMW isoform, with in parallel, a significant gain in lean/muscle mass without changes in fat mass. Together with the described effects of TCZ on qualitative characteristics of lipids, these results argue for a safe CV profile with a favourable impact on the CV burden of RA. However, changes in adiponectin and its HMW isoform are mainly observed at the onset of treatment, and studies with a longer follow-up are warranted.

## Supplementary information

**Additional file 1 **Changes metabolic parameters during the study. (results are given as mean ± SD)(M: month; * paired Student *t* test, #: sensitivity analysis).

## Data Availability

The data used and analysed during the present study are available from the corresponding author on reasonable request.

## Abbreviations

RA: Rheumatoid arthritis
CV: Cardiovascular
csDMARDs: Conventional synthetic disease-modifying anti-rheumatic drugs
bDMARDs: Biological disease-modifying anti-rheumatic drugs
CRP: C-reactive protein
anti-CCP: Anti-cyclic citrullinated peptides
HOMA-IR: Homeostasis model assessment of insulin resistance
TCZ: Tocilizumab
MTX: Methotrexate
LEF: Leflunomide
GC: Glucocorticoids
LMW: Low molecular weight
HMW: High molecular weight
AS: Ankylosing spondylitis
TNFi: TNFalpha inhibitor
NCEP ATP III: National Cholesterol Education Program Adult Treatment Panel III
