# Supplementary material for: Increased high molecular weight adiponectin and lean mass during tocilizumab treatment in patients with rheumatoid arthritis: a 12-month multicentre study
Source: Arthritis Res Ther. 2020 Sep 29;22:224. doi: 10.1186/s13075-020-02297-7 (PMC7523335; doi:10.1186/s13075-020-02297-7)
Supplement: Supplementary file 1 — Additional file 1 Changes metabolic parameters during the study. (results are given as mean ± SD)(M: month; * paired Student t test, #: sensitivity analysis). [file 13075_2020_2297_MOESM1_ESM.docx]

Additional file 1: Changes metabolic parameters during the study

|  | **M0**  **(N = 107)** | **M1**  **(N = 106)** | **M3**  **(N = 104)** | **M6**  **(N =97)** | **M12**  **(N = 77)** | **P***  **M0 vs M6 (N = 97)** | **P***  **M0 vs M12 (N = 77)** | **P#** |
| --- | --- | --- | --- | --- | --- | --- | --- | --- |
| **Glycémia**  **(mmol/l)** | 5.1 ± 1.2 | 5 ± 1.2 | 5.3 ± 1.4 | 5 ± 1 | 4.7 ± 1.3 | 0.5 | 0.2 | 0.14 |
| **Insulinemia**  **(μU/mL)** | 18.6 ± 15.7 | 23.4 ± 43.8 | 19.4 ± 22.9 | 18.8 ± 18.9 | 18.4 ± 12.2 | 0.79 | 0.42 | 0.42 |
| **HOMA-IR** | 4.5 ± 4.4 | 6.2 ± 14.1 | 4.8 ± 5.7 | 4.2 ± 4.5 | 3.7 ± 2.7 | 0.6 | 0.11 | 0.22 |
| **Total Cholesterol (mmol/L)** | 5.2 ± 1.2 | 5.7 ± 1.4 | 5.8 ± 1.3 | 5.7 ± 1.2 | 5.6 ± 1.2 | **<10 ^-4^** | 0.053 | 0.043 |
| **LDL cholesterol (mmol/L)** | 2.9 ± 1 | 3.4 ± 1.2 | 3.3 ± 1.1 | 3.4 ± 0.9 | 3.4 ± 0.9 | **<10 ^-4^** | **0.0018** | 0.0005 |
| **HDL cholesterol (mmol/L)** | 1.6 ± 0.5 | 1.7 ± 0.5 | 1.7 ± 0.7 | 1.7 ± 0.6 | 1.6 ± 0.4 | 0.12 | 0.6 | 0.5 |
| **Total / HDL Cholesterol** | 3.5 ± 1.2 | 3.5 ± 1.1 | 3.6 ± 1.2 | 3.6 ± 1.1 | 3.7 ± 1.1 | 0.9 | 0.18 | 0.15 |
| **Triglycerides (mmol/L)** | 1.4 ± 0.8 | 1.4 ± 0.6 | 1.5 ± 0.9 | 1.4 ± 0.7 | 1.4 ± 0.7 | 0.6 | 0.36 | 0.67 |

(results are given as mean ± SD)(M: month ; * paired Student *t* test, #: sensitivity analysis)
